# Looking for Ted: black trips, “psychedelic” humanism, and silence

**DOI:** 10.3389/fpsyg.2023.1198371

**Published:** 2023-10-18

**Authors:** Michael Cordova

**Affiliations:** The University of Texas at Austin, Austin, TX, United States

**Keywords:** blackness, psychedelic, race, coloniality, archive, humanism, psychedelic science

I look for Ted. A black existence in an archive that has failed him. However, I read not only for failure, but I read for Ted. To meet him, even in all this failure, I wonder if I will fail him. This article focuses on blackness within the psychedelic archive, particularly Grof and Halifax's (1978) *The Human Encounter with Death*. Published in 1977, this book was a product of Grof's research in the early 1970s at Spring Grove Clinic. His work was centered on the emergent scientific inquiry into the effects of psychedelics on “human” test subjects. Grof and Halifax (1978) sought to understand how psychedelics could overcome terminally ill patients' anxiety regarding death. The cultural production of death became critical to their argument, while little cultural scrutiny was given to race, ability, or class. However, the historicity and cultural contextualization of death ran central to their alternative model of engaging what mortality was through psychedelics. My article thus sits with the cultural specificity afforded to psychedelics in conversation with death and imagines the limitations of said specificity when blackness and Otherness are brought into the conversation. I ask the question, “how may we read subjectivity within the psychedelic archive when it comes to a black psychedelic subject?” To answer this question, I examine the implicit anthropological models that ground patient biographies and dissect what I term the “psychedelic humanist model.” Furthermore, in mapping the ways that Grof and Halifax (1978) narrativize Ted, I ponder how an archival engagement with Ted might push against the objectifying trends of psychedelic science. Finally, I piece together what my search for Ted sought to recuperate, but, instead, I found the destabilizing residue of the black trip within the psychedelic archive.

To understand the subject of the text, we must first establish the framework within which the text operates. In particular, what do Grof and Halifax (1978) mean by the titular “human encounter with death”? Who is the human we are reading about? In posing such questions, I take Halifax's training as an anthropologist as a critical point of departure: how is a subject historically rendered in anthropology? Who, then, is afforded this human subjectivity? How does anthropology valorize these normative models through its anthropological subject? As Wynter (2003) notes, the very crux of the political and scientific emergence of Man (which as a term stands for the normative mode of being human) was predicated on the concept's construction by scholars “including centrally those of anthropology” who would force “non-Europeans” to be the “physical referent” (in the episteme of Man) of the “irrational or subrational Human Other” (266). This Man would evolve through what Wynter (2003) calls the “Darwinian Leap,” described in purely secular terms. In these “biologized terms, it was to be the peoples of Black African descent who would be constructed as the ultimate referent of the “racially inferior” Human Other” to Man (266). This is to say that the normative script that Halifax's anthropological methods are working through is sutured to a disciplinary history that can only represent this normative model of humans unless they grapple with and deconstruct the racialized history of humanity as it is normatively known. Halifax and Grof fail to do so, instead abiding by the unquestioned script of Man. Thus, the desired anthropological subject is one that can approach the normative discourse of humanity.

In order to have their subjects occupy this normative humanity, Grof and Halifax's (1978) study neglected elements of blackness and Otherness that are inseparable from their anthropological subject. As a test subject, Ted, then, is afforded his humanity (from which he is normatively excluded) on the terms that a psychedelic experience grants him entry into the position. Thus, the washing away of blackness and Otherness is important to their study, for it pieces together their universalist model as the humanity of Ted seemingly emerges from psychedelic use. This is to say, a new universal model of thought comes into being once blackness and similarly Otherized positions are seemingly erased from the ontological model. We can see this in the way that Ted is “cured” of his Otherness and, through this “cure,” shifts into the cured position of the psychedelic subject. However, the architecture of this racially sanitized and seemingly curative subjectivity is built upon a shaky foundation of a universalizable engagement with death.

This desire to undo the normative, empirical prescriptions of death is hinged on the fabrication of our current empirical mappings of death. For Grof and Halifax (1978), death is a problem because of the way it is normatively presumed to be an endpoint of the state of being in the West. This illuminates why their reading is interested in “compar[ing] the situation of a person facing death in contemporary Western civilization with that of individuals in ancient cultures or from preindustrial countries… [since m]ost non-Western cultures have religious and philosophical systems, cosmologies, ritual practices, and certain elements of social organization that make it easier for their members to accept and experience death” (2). Though an arguably reductionary comparison, they seek to mobilize disparate cultural definitions of death to challenge the normative prescription of death in the West. This, according to Grof and Halifax (1978), would aid in overcoming the anxiety around death, which is arguably a product of Western empiricism that offers no answer to what death is. This gap then leaves their work as a means of rethinking what death is through non-normative means: psychedelics. “Psychedelic drugs,” they maintain, “have made it possible to develop a new understanding of the symbolic death-rebirth process that occurs in shamanic initiation, rites of passage, temple mysteries, and some schizophrenia episodes. Deeper phenomenological analysis shows that the extended map of the human unconscious derived from LSD research is indeed applicable to all these situations” (Grof, p. 175). However, in a tenuous line, Grof and Halifax (1978) are theorizing that psychedelic science might provide the empirical foundation for a cosmology beyond our normative empiricist life-death order. In other words, death is not the end but merely an entryway into another way of being human—a seemingly better way due to its curative qualities. Yet, their work suggests that these “ancient” claims must be “reformulated in modern terms” through the empiricism of phenomenological analysis of psychedelic experiences (175).

Thus, the terminally ill patient becomes the psychedelic subject that will (re)map these potential cosmologies that speak toward a hereafter. Importantly, this mapping process also appears to redefine the concept of the human, as observed in Grof and Halifax's (1978) rigorous contestation of the purely secular model of Man that defines the present human within their work. Instead, they turn toward empiricism (or, better termed, reason)^1^ yet yearn to sit with the spiritual as well, hence their common collapsing of “non-normative spiritual experiments” by patients into Buddhist or Hindu readings (67, 79, 83, 93, 101). Though this common practice in their work did not limit their test subjects to a particularly Buddhist or Hindu narrative, it did foreclose their non-normative spiritual explorations—as seemingly arrived at through psychedelic experiences—entirely into Grof and Halifax's (1978) “new” humanism. In effect, this “new” human model they yearned for was a reasoned yet spiritual model of the human—a psychedelic humanism. A movement of the human away from the normative precepts of contemporary Western, purely materialist empiricism toward an apparently unstable humanism that dared to accept not just a material way of being but also a spiritual way of being human, which became accessible through psychedelic experiences.

For all its seeming opposition to normative Western narratives, this model essentially replicates the secular-religious framework of Man that underpins liberal humanism. As Wynter (2003) would describe it, this is a “hybrid religio-secular” (277) conception of Man, whose “choice [was] that of either growing downwards into the lower natures of brutes or responding to the Creator's call to grow “upward” to “higher” and “divine” natures” (287). Though not an exact model, the similar model speaks to psychedelic humanism's curative yearning for a metaphysical frontier found through the material plane of being. This is to say, psychedelic humanism reflects the Wynterian “hybridly religio-secular” in its use of psychedelic subjects to map out a spiritual being while simultaneously instrumentalizing the material plane as a tool for accessing this spiritual elsewhere. This elsewhere becomes central to Grof and Halifax's (1978) project, for this is where marginalized subjects (particularly disabled patients) serve their use-value for the benefit of science, especially Ted. Thus, their apparently human condition afforded through psychedelic use continues the practice of the Other, becoming the defining lack for the human of the text. Their entry into “humanity” is a mere ploy to map out the limits of the psychedelic experience and thus prove that psychedelics can be an empirically viable cure for anxiety around death.

With this humanist-defining mechanism in place, we must understand the archival position that this research occupies, for it speaks toward a genealogical necessity in the research of psychedelic science. I must acknowledge that Grof and Halifax's (1978) work is not at the forefront of psychedelic science and its present discourse. Nevertheless, the very discipline, as it is known today, is built off of the research done at Spring Grove Clinic, just as it is built off the backs of so many other psychedelic science institutions and projects. In their discussion about the Spring Grove Clinic, Yensen and Dryer (1992) argue that psychedelic scientists “must use the insights available from past efforts in this culture and others to develop” contemporary research efforts (21). The research at Spring Grove Clinic's cultural influence carries sway since Rick Doblin was trained and influenced by Grof (Endwell Project, 2021). Notably, the Multidisciplinary Association of Psychedelic Studies (MAPS), where Doblin is the executive director and its founder, doled out $12 million in the 2019–2020 fiscal year for psychedelic research (Christiansen et al., 2020). However, this is small compared to the companies investing hundreds of millions into the research and development of psychedelic drugs (Psychedelic Alpha, 2022). MAPS has been a cultural attaché for psychedelic research and therapist training. In thinking through these terms, it is critical to view the work done in *The Human Encounter with Death* not in a vacuum but as a lived cultural artifact imbricated in the ongoing discourse of psychedelic science/therapy. This can be easily noticed in contemporary studies that have inherited the same central questions on the influence psychedelic experiences can have on anxiety toward death that Grof and Halifax (1978) originally focused on.^2^ Moreton et al. (2019) even acknowledge a direct line as they state how their work “echoes the claims of many early psychedelic researchers such as Pahnke” (28). Pahnke et al. (1970) directed the Spring Grove Clinic from 1967 to 1971 and worked alongside Grof on studies related to anxiety toward death.^3^ Subsequently, while Grof and Halifax's (1978) text is not contemporary psychedelic science, it speaks to the discursive focus of a particularized time that continues to influence the field, and it manages to explicitly fold blackness (through the figure of Ted) into the complex conversation of psychedelics. Thus, in acknowledging the psychedelic archive, we are not viewing these texts as the end all be all but rather as emergent conversations that establish and set foundational discursive norms. As such, the archive provides us a mode of thinking with these emergent discourses without acting as if they came out of thin air. Moreover, we are provided the space to think with particular ruptures and movements that give rise to approaches or the lack of approaches that have been inherited over time.

With this archival acknowledgment in mind, I turn to Ted to examine his influence on our psychedelic conversation. What we come to find is that Ted occupies a particularized role that “functions to index the limit of science” due to his “blackness” (Warren, 2018, p. 110–111). According to Warren (2018), it is through nothingness that science can even function, but it is by avoiding/overcoming nothingness (thus blackness) that science fundamentally works (110). As the very “function of black(ness) is to give form to a terrifying formlessness (nothing)” (5), the very idea of nothing within our epistemic structure, as organized through humanism, requires a lacking position to structure what it means to be and who is afforded being. Arguably, Warren's (2018) nothing is the space of the Other that Wynter (2003) discusses. It is a lacking space that is not being (human). Through these theoretical frameworks, we find that Ted is not himself but the narrative scripts of his seeming inhumanity and the reparative ground that psychedelics offered him to become human. This tale is curated through the careful mediation of Grof and Halifax (1978) as scientific and anthropological facilitators.

Ted was “a twenty-six-year-old Afro-American suffering from an inoperable cancer of the colon” (69). Grof and Halifax (1978) described him as having limited education and “fairly open to a religious worldview. Communication in his family was disoriented and complicated and required much psychological work” (63–64). He had a wife named Lilly and three kids.

His entire childhood was characterized by severe emotional deprivation and outright physical abuse. He lost both parents at the age of 3 and spent several years in various orphanages. Finally, he ended up in the house of his uncle and aunt, who became his foster parents. In their home, he suffered much rejection and cruel emotional and physical abuse. During his childhood and adolescence, Ted was involved in minor antisocial activities, had frequent fistfights with individuals in the framework of street gang skirmishes, and liked rough entertainment. Later, he enjoyed his involvement in the war, where his aggressive tendencies found a socially approved channel. While being married to Lilly, he was extremely jealous of her but had strong tendencies toward extramarital affairs himself (71).

This is Grof and Halifax's humanizing description of Ted which implicitly sutures him to blackness through his violence, broken home, hypersexuality, and vulnerability. It is an exclusionary description that posits his inhumanity as an anthropological subject of study, but it will be through psychedelics that his humanity can emerge and reimagine death.

Ted becomes a site of excess for Grof and Halifax (1978), for if he can be brought into humanity, then perhaps these curative and exploratory models they are formulating have a use. In this article, Grof and Halifax's (1978) descriptions of other patients begin to paint the “Chain of Being” (Wynter, 2003, p.300). Ted is compared to the “full aware[ness]” of other text subjects such as Matthew, who was “a physician,” or Susanne, the “attractive, sensitive, and intelligent woman… [who] was involved in the study of psychology,” or Joan who “was a forty-year-old housewife and mother of four children” (63, 83, 93). Respectively, each of these people abides by the same narrative scripts that bind Ted. Each of these patients who are not Ted displays a site that can be returned to, as their lives were seemingly success stories until stifled by disability and mortality. Their narratives speak to the overrepresented chain of humanity that defines this text. Each biography is interlinked with the other to imagine the potential ways psychedelics can cure anxiety about death. This chain thus places Ted at the very bottom ring. It is also important to note that none of the other patients are denoted as “white” or “European-American” or any other racialized group, for this is the expected normative position they would occupy. Ted is also described as “in many respects on the opposite side of the spectrum” when compared to Matthew (63). Though a seemingly insignificant fact, these omissions perpetuate the greater script that the text is working toward. If Ted can be cured, then everyone else can be cured, for he is the bottom ring of the chain of humanity.

Interestingly, another test subject, Jesse, is mobilized in close approximation to Ted. Jesse's presence speaks to a criticism of class; he is described as an “almost illiterate” (64) man who “changed jobs several times, and because of his limited education, did not reach high positions in any of them” (80). To read Ted also requires us to sit with Jesse and read him as an object of class analysis that psychedelic science, in this instance, intends to cure. Thus the precarious racial and/or class conversation in Grof and Halifax's (1978) text reflects an ontic presumption of inability, which is further perpetuated by the embodied disability of inoperable cancers. The terminal conditions that these psychedelic subjects occupy speak to the precarity of this exploration, in which only expendable bodies are put into the position of explorer within the emergent scientific discourses of the time. While many folks used psychedelics in the 1970s, the scientific model was constructed as curative toward madness, addiction, and anxiety, eventually leading to death (as seen in Spring Grove). Thus, these structurally negated people on the ontological margin, with Ted's blackness situated as this margin's limit, and structure the project in the space of the Other. Through careful tabulation, deciphering, and integration, this map of death can be set into an abstract model of humanism that Grof and Halifax (1978) work through. Thus, this emphasizes the potential for an utter abstraction from materiality, which these psychedelic biographies ground.

The narrative structure in Grof and Halifax's (1978) text cared for nothing more than the use value that Ted afforded through his blackness, but what of my search? Ultimately, I ran up short, not because Ted never existed, but because the search for Ted was the search for nothing [as Warren (2018) would argue]. Thus, the normative options when faced with this archival lack (or nothingness), according to Nyong'o (2019), often push critics to piece together the “exploitation” that defines the text or toward the hope of “restor[ing]” the subject of the text to some form of being (48–49). In other words, these models postulate totalizing narratives that, within themselves, seemingly foreclose the frame as utterly denying the subject, but what if the subject within the framework is not denied? What is the eruptive capacity of what might rest within the framework itself? As Macharia (2016) warns, “Recovery and representation can never be easy: minefields abound, and one attempts to minimize the damage one will cause” (p. 501). This incessant desire to either move past the violated subject or romanticize them is a dangerous game, for we run the risk of unthinking the damage done and instantiating foreclosure on new, seemingly ethical terms. Instead, as Macharia (2016) suggests, even in the violent potentials of careful analysis, an attempt must be made to piece together a relationship through the incommensurability and ineffability of the archive. Thus, in my reading of Ted, I tarry with the very real potential of my own mode of objectification. As the desire for restoration only makes another object out of a supposedly restored subject, “Instead of the search for an object that leads to a subject, the scholar's search should be for a subject effect: a ghostly afterlife or a space of absence that is not empty but filled” (Young, 2017, p. 3). Working in the vein of Anjali Arondekar's archival theorizations, Young (2017) argues for an opaque model of reading that moves against reinventing violence toward a model that can grapple with “the disjunctures, chasms, and nodes of connection between different historically located fields of knowledge that can help us more fully flesh out the afterlife of black diasporic subjects” (3–4). This extension leads to a broader conversation within psychedelic science, which focuses on blackness, Otherness, and structural violence. In this context, we must move away from merely removing or adding these devalued narratives. The former is the psychedelic humanist model that Grof and Halifax (1978) employed, while the latter serves as the recent impetus in psychedelic scholarship toward inclusion^4^, which has inherited a similar valence of violence to the former, as argued by Macharia (2016), Nyong'o (2019), and Young (2017). An opaque reading mode moves against these subject-restoring projects, instead engaging silence for what it offers. It challenges researchers to meet blackness within the object position, which is normatively avoided due to the messy history of colonial violence that has long defined modernity. Within this meeting space, we can witness how Ted's blackness is a mere afterthought. His Otherness is a useful tool for psychedelic research. His humanity is wholly predicated on faulty universalism. If we begin here, we begin to realize the violent model of reading the trip that is normative for blackness as it simultaneously erases blackness. A model that bars this existence and merely uses it like one might be a psychedelic substance.^5^

In reading against this normative trip, I offer up the black trip as a reparative alternative within the psychedelic archive: a model that dares to sit with nothingness, difference, incommensurability, excess, and all the other forms that we might read blackness through. However, this particular model, situated within the psychedelic archive, positions blackness not as a thing to be overcome, as Grof and Halifax (1978) implicitly argue, but instead as a position to be sat with and reimagined even in its violated place within the archive. The black trip destabilizes the normative terrain it occupies through its devalued presence. In effect, via the denied position in which Ted is placed, we cannot necessarily reread him into being in the way that suits us. Instead, our readers must accept this violence and move with it. It must accept the gap of his being and position nothingness as a generative site of thought. Thus the black trip puts into question the very normative framing of psychedelic science through its humanist model and examines the limits of its uses for all patients. In effect, the black trip is a destabilizing model of rereading the psychedelic archive, as it dares to sit with the violence rather than merely acknowledge it. It yearns to know how, within the history of science and psychedelics, it emerges the way it does and affects its discursive productions. To this end, the black trip sits with the residue of people like Ted, Jesse, and all the marginalized psychedelic subjects and does not recover them but merely acknowledges the violence that will never be totally known. Once carefully deconstructed, these violences begin not just to remap but to undo the normative methods. The black trip then dares us, as researchers, to acknowledge our limitations yet continue to work toward and with the violated. The black trip does not offer a solution, but it offers a mode of thinking through the ineffable violations and continual reimaginings that psychedelic science mobilizes. I may not have found Ted, nor will I truly ever, but I sit with him now. He is nothing, but we must not fear nothing, it is a scarily generative way to exist within humanism's violent epistemology.

## Author contributions

The author confirms being the sole contributor of this work and has approved it for publication.

## Footnotes

^1^For a more thorough engagement with antiblackness, empiricism, and reason, please see Bruce's (2020) *How to Go Mad Without Losing Your Mind: Madness and Black Radical Creativity*. Bruce (2020) maps out the rampant epistemic violence that empiricist and reasoned epistemologies sustain, especially through their intentioned exclusion of black and otherized existences (p. 4).

^2^See Griffiths et al. (2016), Sweeney et al. (2022). Griffiths also cites Grof's 1973 qualitative study on psychedelics and anxiety toward death, continuing to validate the influence of this research to contemporary psychedelic science (Grof et al., 1973).

^3^See Pahnke et al. (1970), Kurland et al. (1972).

^4^See George et al. (2019), Williams and Labate (2019), Morales et al. (2022) for recent discursive examples of inclusivity within the psychedelic science archive. To note, this article is not arguing against treating black patients with psychedelic drugs, but rather the article is suggesting that normative methodological approaches within psychedelic science should expand past recuperative models of inclusion toward engaging the structural history of silence that black patients have and will continue to endure. It is in reckoning with this silence that we can come to remap the normative methodological terrain that defines the meeting of blackness with the psychedelic.

^5^This argument of blackness as being consumed like a psychedelic substance is in direct lineage to Walter's (2021) notion of ‘ontological envelopment. Walter (2021) particularly focuses on the role of addiction, narcotics, and blackness through his formulation of “Intoxicated blackness.” Central to his thesis is that “[the] intoxicated black being in turn becomes toxin, where the addict takes on the role of the drug” (9). Further on, he argues, through his notion of intoxication, that blackness, due to its ontologically enveloped position, “is both intoxicated and… an intoxicating agent” (19). Thinking through this double-bind is key to my argument on the black trip, because blackness structurally occupies an object position of foreclosure—Ted showing this. Yet simultaneously this object position sustains a means of being read/engaged with in a way that can speak toward the material measure of violence that sustains coloniality, humanism, and the West. Thus the black trip is in line with Walter's (2021) arguments on intoxication and addiction, but refitted toward a psychedelic context.
